# Noninvasive vagus nerve stimulation and morphine transiently inhibit trigeminal pain signaling in a chronic headache model

**DOI:** 10.1097/PR9.0000000000000881

**Published:** 2020-12-17

**Authors:** Lauren E. Cornelison, Jordan L. Hawkins, Sara E. Woodman, Paul L. Durham

**Affiliations:** Missouri State University, Center for Biomedical and Life Sciences, Springfield, MO, USA.

**Keywords:** Trigeminal, Sensitization, Vagus nerve, Chronic headache, Sleep deprivation, Opioid

## Abstract

**Introduction:**

Chronic headache conditions are characterized by persistent sensitization of the trigeminal system, which involves dysfunction of descending pain modulation. We previously reported that noninvasive vagus nerve stimulation (nVNS) inhibits trigeminal nociception in models of episodic migraine through a mechanism involving enhanced serotonergic and GABAergic descending pain signaling.

**Objectives:**

The analgesic effectiveness of nVNS and morphine were investigated in an animal model of chronic headache mediated by the combination of the 3 migraine risk factors of neck muscle tension, paradoxical sleep deprivation, and pungent odors.

**Methods:**

Sprague–Dawley rats were injected with complete Freund's adjuvant in the trapezius and sleep deprived for 1 night to promote trigeminal sensitization. After 7 days, animals were exposed to a pungent odor, and mechanical nocifensive head withdrawal responses were determined using von Frey filaments. Beginning on day 3 after odor exposure, animals were treated daily with either nVNS or morphine for 7 days.

**Results:**

Exposure of animals sensitized by neck inflammation and sleep deprivation to a pungent odor resulted in a prolonged state of trigeminal nociception. Daily administration of nVNS or morphine significantly repressed the nocifensive response; however, cessation resulted in a return to heightened pretreatment nocifensive levels.

**Conclusions:**

The combination of reported migraine risk factors promotes a state of sustained trigeminal hypersensitivity characteristic of chronic headache. Daily nVNS was similarly effective as morphine in inhibiting nociception and may represent a safer, opioid-sparing therapeutic option for other chronic pain disorders involving sensitization of the trigeminal system by promoting descending pain modulation.

## Abstract 2

Noninvasive vagus nerve stimulation suppressed persistent trigeminal nociception in a chronic headache model similarly to morphine and may provide a safe, nonaddictive abortive therapy for chronic headache.

## 1. Introduction

Prevalent and disabling orofacial pain conditions, including migraine and temporomandibular disorders, are associated with development of peripheral and central sensitization of the trigeminal system.^14,50^ Migraine is a common painful neurologic disorder classified as either episodic or chronic migraine based on the International Headache Society criteria on attack frequency.^26^ Chronic migraine, which causes greater disability than episodic migraine and affects ∼2% of the general population, is characterized as 15 or more headache days per month for at least 3 months with 8 or more days per month meeting migraine criteria.^60^ A main symptom associated with migraine is throbbing headache, which persists for up to 72 hours and involves sensitization and activation of primary and secondary trigeminal nociceptive neurons.^22^ Other commonly reported migraine symptoms are nausea, vomiting, and heightened sensitivity to light and sound.^43^ Many migraine risk factors and triggers have been identified including stress,^35,38^ bright or flickering light,^25^ pungent odors,^52^ and changes in weather.^34^ In addition, chronic migraine is associated with other comorbidities such as musculoskeletal pain, sleep disorders, and psychiatric disorders.^12,19,61^ Neck muscle pain and sleep deprivation, which are associated with migraine pathology,^13,20,61^ cause dysfunction of the descending pain inhibition pathway leading to the development of central sensitization and pain amplification.^2,33^ Migraine pathology involves a reduction in descending pain modulation, sustained trigeminal sensitization, and a lowered threshold to sensory stimuli.^11,14^ Chronic migraine has fewer effective therapeutic options than episodic migraine and is associated with more comorbid conditions and greater disease burden.^10,16,36^ However, noninvasive vagus nerve stimulation (nVNS) may offer a novel, safe, and effective method for treating chronic migraine and other chronic orofacial pain conditions.^63^

In a recent study, nVNS inhibited trigeminal nocifensive response to mechanical stimulation in a preclinical model of episodic migraine through a mechanism that involved stimulation of the serotonergic and GABAergic inhibitory pain pathway.^15^ Based on the efficacy and safety results from multiple clinical trials, nVNS has FDA clearance for the treatment and prevention of cluster headache and migraine.^21,44,58^ The ability of nVNS to inhibit pain signaling is associated with changes in the expression of proteins implicated in peripheral and central sensitization^24^ and facilitation of descending pain modulation.^27^ Our results demonstrated that the inhibitory effect of nVNS is mediated through activation of GABAA, 5-HT3, and 5-HT7 receptors,^15^ which are activated by morphine.^4,5^ Although the use of morphine and other opioids have traditionally been used in emergency departments as an effective abortive treatment of migraine,^17,53^ their continued use is not recommended given their negative side effects including medication overuse headache and a high potential for dependency and misuse.^8^

A major goal of this study was to determine if the combination of the reported migraine risk factors including neck muscle tension, sleep deprivation, and strong odors could lead to a sustained hypersensitized state of trigeminal nociceptive neurons characteristic of chronic headache. The ability of nVNS or morphine to treat and possibly stably reverse the prolonged level of nociception in this chronic headache model was also investigated. The main findings were that although each individual risk factor alone did not cause trigeminal nociception, their combination resulted in an enhanced level of nociception that was inhibited by daily treatment with nVNS or morphine.

## 2. Methods

### 2.1. Animals

Sixty-three adult Sprague–Dawley male rats (200 g–300 g) were purchased from Missouri State University's Central Management Breeding Colony (Springfield, MO) and allowed to acclimate for 1 week to facility conditions. Animals were purchased as a series of independent cohorts of 6 to 8 animals, and after acclimation were randomly assigned to experimental groups. Animals were housed individually in plastic rat cages with aspen chip bedding and unrestricted access to both food and water in a room with 12 hour/light dark cycles. All protocols were approved by Missouri State University's Institutional Animal Care and Use Committee and conducted in compliance with the Animal Welfare Act, National Institutes of Health, and ARRIVE Guidelines. A concerted effort was made to minimize suffering and the number of animals. The attending veterinarian provided guidance on appropriate morphine dosing.

### 2.2. Inflammation of trapezius

To induce prolonged neck muscle inflammation, some animals were placed under 3% isoflurane in oxygen and received injections of complete Freund's adjuvant (CFA, Sigma-Aldrich, St. Louis, MO; 1:1 in 0.9% sterile saline) into the upper trapezius muscles as previously described.^15,24,46^ A total volume of 100 µL was injected with the right and left muscles each receiving 5 microinjections of 10 µL. As a vehicle control, some animals received similar muscle injections with 0.9% sterile saline. Animals recovered in their cages and were monitored for normal grooming, feeding, and movement.

### 2.3. Sleep deprivation

Paradoxical or rapid eye movement sleep deprivation was accomplished based on a modified version of the platform model.^30,39^ To study the effect of 24 hours of sleep deprivation, some animals were placed in modified cages containing a 3 × 3 × 3 inch platform surrounded by room temperature water. The animals could sit, crouch, and achieve non-rapid eye movement (REM) sleep on the platform. Because of the requirement of postural muscle atonia during REM sleep, animals contacting the water are awakened. Animals in the modified cages were placed in a rodent incubator for 24 hours. Animals in normal bedding served as naive control animals. After sleep deprivation, animals were returned to fresh-bedded cages for 2 hours before nocifensive assessment.

### 2.4. Exposure to pungent odor

Eight days after neck muscle inflammation and/or REM sleep deprivation, animals were exposed for 10 minutes to the pungent odor from an oil extract prepared from raw California Bay Laurel tree leaves (Spice World, Seattle, WA) to cause activation of trigeminal nociceptors.^15,24,46^ Briefly, animals were placed in a box attached to a tube with oxygen flowing through at a rate of 2 L/min. A cotton swab containing 20 µL of extracted oil was placed in the tube such that animals could not directly contact it. After exposure, animals were returned to their normal cages for 2 hours before nocifensive testing.

### 2.5. Treatment with noninvasive vagus nerve stimulation or morphine

The procedure for nVNS was performed essentially as described previously using a modified VNS device (electroCore, Basking Ridge, NJ).^15,24^ Three days after exposure to the pungent odor, animals were lightly anesthetized with isoflurane, and the stimulator electrodes were coated in electrolyte gel and placed on a hairless region over the vagus nerve. A 1 ms pulse of 5 kHz sine waves, repeated at 25 Hz, for 2 minutes was administered and was followed 5 minutes later by a second 2-minute stimulation, which is identical to the paradigm used in humans. Animals were treated daily for 7 days and after treatment recovered in their cages. Similarly, 3 days after odor exposure, some animals received morphine initially at 10 mg/kg for 2 days, then 15 mg/kg for 2 days, and finally 20 mg/kg for the remaining 3 days (morphine sulfate salt pentahydrate, Sigma-Aldrich) subcutaneously twice a day for a total of 7 days. Our experimental paradigm for morphine dosing was based on a previous study in which persistent peripheral inflammation was found to attenuate analgesic tolerance in male rats.^18^

### 2.6. Nocifensive behavior studies

**Figure 1.**
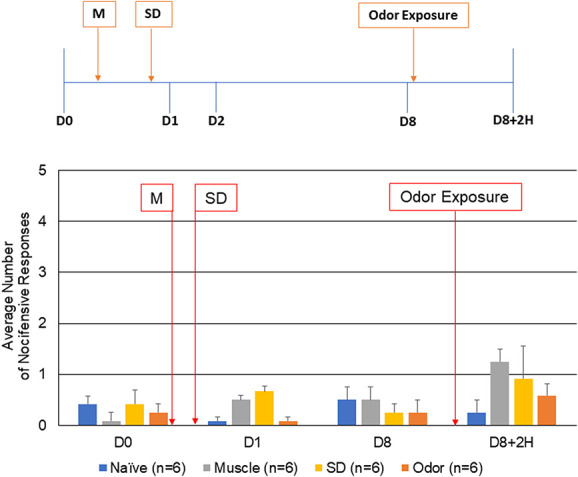
Neck muscle inflammation, sleep deprivation, or pungent odor alone do not promote development of a sensitized state of trigeminal nociceptors. The average nocifensive head withdrawal response ± of 5 applications to each side to mechanical stimuli are reported. Some animals were left untreated (naïve), some were only injected with CFA (muscle, M), some were only REM sleep deprived for 24 hours (SD), and some were only exposed to the pungent extract from California Bay Laurel leaves (odor). Animals were tested for mechanical sensitivity basally (D0), 1 day after muscle injections or after overnight REM sleep deprivation (D1), 8 days after muscle injections/7 days after REM sleep deprivation (D8), and on day 8, 2 hours after exposure to odor (D8+2H). CFA, complete Freund's adjuvant; REM, rapid eye movement.

**Figure 2.**
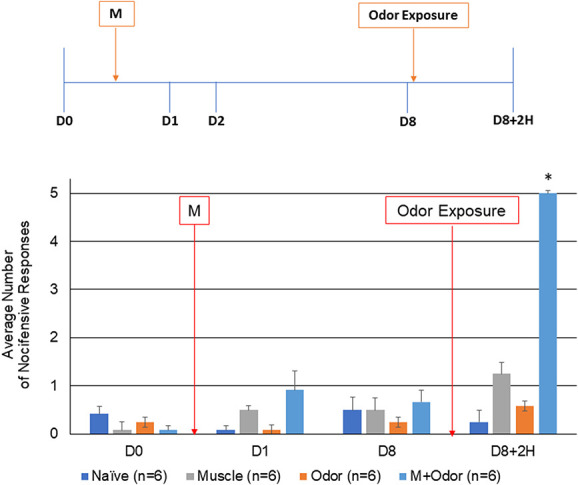
Exposure to pungent odor causes an allodynic response in animals with neck muscle inflammation. The average nocifensive head withdrawal response ±SEM from 5 applications to each side to mechanical stimuli are reported. Some animals were left untreated (naïve) for 8 days. Some animals were only injected with CFA or exposed to the pungent extract from California Bay Laurel leaves (muscle, odor, respectively), whereas others were injected with CFA 8 days before exposure to pungent odor (M + odor). Nociceptive responses were determined on day 0 (basal), at day 1, and day 8 after CFA injections, and 2 hours after odor exposure. *P < 0.05 compared with naïve. CFA, complete Freund's adjuvant.

**Figure 3.**
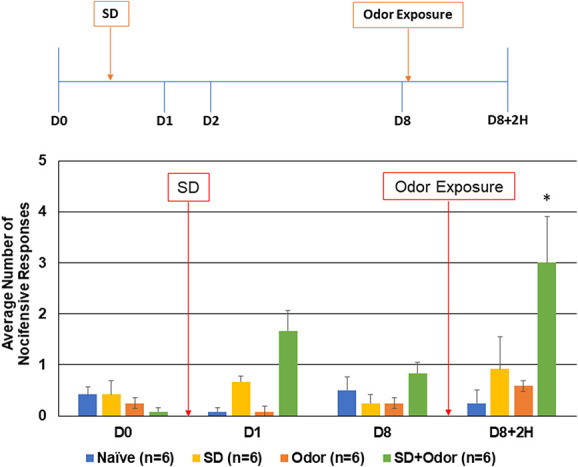
Exposure to pungent odor mediates mechanical allodynia in animals deprived of REM sleep for 1 night. The average nocifensive head withdrawal response ±SEM of 5 applications of mechanical stimuli to each side are reported. Some animals were left untreated (naïve) for 8 days, whereas some animals were deprived of REM sleep for 1 night (SD) and some exposed to the pungent extract from California Bay Laurel leaves (odor). Some animals received both sleep deprivation followed by odor exposure 8 days later (SD + odor). Nociceptive responses were determined on day 0 (basal), directly after sleep deprivation, as well as 8 days after basal testing and 2 hours after odor exposure. *P < 0.05 when compared with naïve. REM, rapid eye movement.

**Figure 4.**
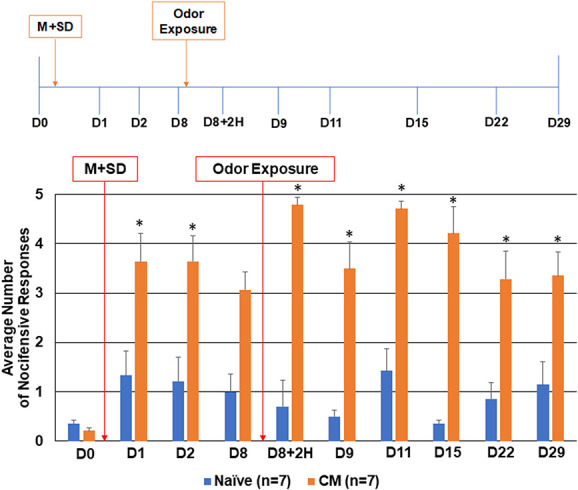
Exposure to pungent odor causes a sustained allodynic response in animals when preceded by neck muscle tension and 1 night of REM sleep deprivation. The average nocifensive head withdrawal response ±SEM of 5 applications to each side to mechanical stimuli are reported. Some animals were left untreated (naïve), whereas some animals received CFA injections into the trapezius, were subjected to 1 night of REM sleep deprivation, and were exposed to the pungent odor 8 days later (CM, chronic model). Nociceptive responses were determined on day 0 (basal), directly after sleep deprivation and again the next day (D1, D2), directly before and after odor (D8, D8+2H), and 1, 3, 7, 14, and 21 days after odor exposure (D9, D11, D15, D22, and D29). *P < 0.05 when compared with naïve at that time point. CFA, complete Freund's adjuvant; REM, rapid eye movement.

**Figure 5.**
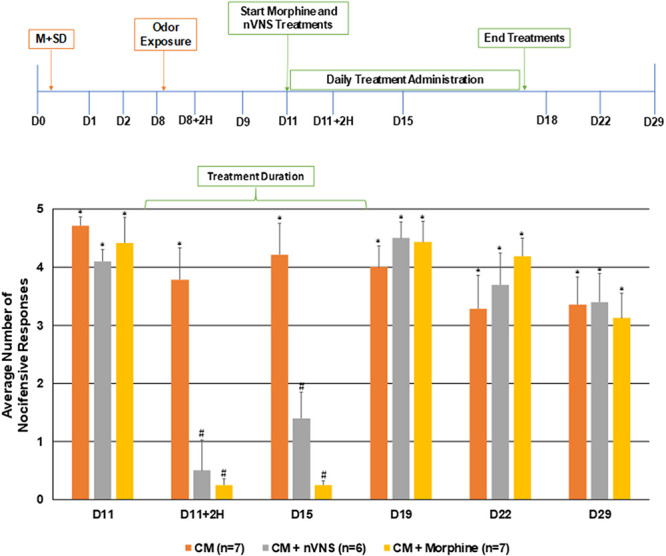
Noninvasive vagus nerve stimulation and morphine transiently inhibit trigeminal nociception to mechanical stimulation in a model of chronic orofacial pain. The average nocifensive head withdrawal response ±SEM of 5 applications to each side to mechanical stimuli are reported. Animals were tested for mechanical sensitivity directly before and after the first treatments with nVNS or morphine (D11, D11+2H), 4 days after treatments were started (D15), and 1 day, 4 days, and 11 days after treatments had concluded (D19, D22, and D29). *P < 0.05 compared with naïve at that time point; #*P* < 0.05 compared with the chronic model (CM). nVNS, noninvasive vagus nerve stimulation.

All behavioral studies were performed between the hours of 6 am and 12 pm. Before testing, animals were acclimated to the Durham Animal Holder (Ugo Basile, Varese, Italy) for 5 minutes on 3 consecutive days. To reduce false positive responses during assessments, animals were conditioned to a mechanical stimulus by gently probing the facial area in a manner similar to behavioral testing with a von Frey filament. This method measures deep musculoskeletal pain responses rather than cutaneous, reflexive defensive responses, and hence, higher weight filaments were required to test nociception. Baseline assessments were conducted 48 hours after acclimations were completed. Mechanical nocifensive thresholds were determined in response to a series of calibrated von Frey filaments (26, 60, 100, 180 g) applied in increasing force to the cutaneous region of the skin that overlies the masseter that is innervated by the V3 branch of the trigeminal nerve.^15,24^ Nocifensive withdrawal reaction, which was defined by a head withdrawal observed before the bending of the filament, was measured by 2 scientists that were blinded to the experimental conditions, with one scientist conducting testing whereas the other scientist retrieved deidentified animals from their home rooms in a random order, then remained in the testing room to verify and record responses for each animal. Each filament was applied 5 times on each side of the face to obtain an average number of reactions. Additional measurements were taken as outlined by timelines (Figs. 1–5). All behavioral timepoints were measured by the same scientist for a given cohort. The 100 g filament was selected for analysis as it elicited a response less than half of the time during basal readings, whereas the next filament, 180 g, elicited a response nearly 100% of the time in untreated animals. Nocifensive reactions are reported as the average number of reactions ±SEM.

### 2.7. Statistical design and analysis

Statistical analysis of the nociceptive behavioral results was performed similarly to previous studies.^15,24^ Using G-Power to conduct a power analysis and using a power of 0.80 at α = 0.05, the minimum sample size to detect expected main effect and interactions required at least 6 animals per group. Data were initially evaluated for normality using a Shapiro–Wilk test. Behavioral data were found to be non-normal (*P* < 0.05), so nonparametric statistical tests were applied. To determine if nociception was different across all groups, a Kruskal–Wallis test was performed. For the single risk factors (Fig. 1), no significant differences were found between groups (df = 3; *P* > 0.05 for each timepoint). When 2 or more risk factors were included (Figs. 2–4), significant results were found between groups (*df* = 7; *P* < 0.05 for several timepoints). In addition, when animals treated with nVNS or morphine were compared with naïve and untreated animals with 3 risk factors (Fig. 5), significant results were found between groups (*df* = 3; *P* < 0.05 for several timepoints).On reaching a significant result, a Mann–Whitney *U* test with a Wilcoxon's W post-hoc test was performed to determine if there were pairwise differences in nociception between groups at each time point. Statistical analysis was performed using SPSS 24 (IBM), and changes were considered significant if *P* < 0.05.

## 3. Results

### 3.1. Individual risk factors did not cause changes in nociception

Initially, the level of trigeminal nociception to mechanical stimulation was determined with the use of von Frey filaments in animals subjected to a single reported migraine risk factor (Fig. 1). The average number of nocifensive head withdrawals to mechanical stimulation was less than 1 response of 5 applications at the basal time point for all experimental conditions with no significant differences between any groups tested (naïve: 0.42 ± 0.16, muscle: 0.08 ± 0.09, sleep deprivation (SD): 0.42 ± 0.29, and odor: 0.25 ± 0.19, *P* = 0.161). The nociceptive response for all conditions including animals that received CFA injection or sleep deprivation were similar to naïve levels at day 1 (naïve: 0.08 ± 0.09, muscle: 0.50 ± 0.34, SD: 0.66 ± 0.12, and odor: 0.08 ± 0.09, *P* = 1.00) and day 8 (naïve: 0.50 ± 0.28, muscle: 0.50 ± 0.20, SD: 0.25 ± 0.19, and odor: 0.25 ± 0.27, *P* = 0.535). As reported in a previous study, injection of saline, which was used as the vehicle for CFA, did not cause a significant change in the average number of nocifensive responses.^15^ Similarly, no significant change in the average number of nocifensive responses was observed 2 hours after exposure of day 8 animals to the pungent odor alone (naïve: 0.25 ± 0.28, muscle: 1.25 ± 0.85, SD: 0.91 ± 0.70, and odor: 0.58 ± 0.26, *P* = 0.240). Thus, exposure to a single risk factor did not cause enhanced trigeminal nociception.

### 3.2. Muscle injections and sleep deprivation lowered activation threshold for odor

To determine if each of the risk factors could be lowering the activation threshold of trigeminal nociception, animals subjected to either muscle inflammation or sleep deprivation were exposed to the pungent odor. As seen in Figure 2, exposure of animals with ongoing neck muscle inflammation to the pungent odor resulted in a significant increase in the average number of nocifensive responses to mechanical stimulation in the orofacial region 2 hours later (4.25 ± 0.82, *P* = 0.006, U = 0.000, Z = −3.207). However, in agreement with previous studies,^15,24^ the level of nociception was transient, returning to near naïve levels 24 hours after exposure (data not shown). In a similar manner, one night of paradoxical sleep deprivation was sufficient to mediate trigeminal sensitization to the pungent odor leading to a significant (3.00 ± 0.99, *P* = 0.009, U = 0.000, Z = −3.000), yet transient (data not shown) increase in the nocifensive response 2 hours after odor exposure (Fig. 3).

### 3.3. Combination of risk factors leads to sustained increase in nociception

To determine if a combination of known migraine risk factors could lead to a sustained state of trigeminal nociception, animals were subjected initially to neck muscle inflammation followed directly by one night of paradoxical sleep deprivation before being exposed to the pungent odor 8 days later. As shown in Figure 4, animals with neck muscle inflammation and sleep deprivation exhibited a significant increase in the average number of nocifensive responses on days 1 and 2 (4.5 ± 0.42, *P* = 0.017, U = 6.000, Z = −2.388; 3.62 ± 0.52, *P* = 0.038, U = 8.500, Z = −2.074). By 8 days after exposure to both risk factors, the level of nociception remained elevated over naive levels but was no longer statistically different (3.07 ± 0.36, *P* = 0.097, U = 11.000, Z = −1.750). Nociceptive levels continued to gradually decrease for the duration of testing, reaching naïve levels at 21 days (1.0 ± 0.18, *P* = 0.295, U = 13.000, Z = −1.174; data not shown). In latent-sensitized animals on day 8, exposure to the pungent odor resulted in a significant change in trigeminal nociception 2 hours after exposure (4.78 ± 0.15, *P* = 0.011, U = 5.000, Z = −2.664). This increase was still seen 1 day (3.5 ± 0.54, *P* = 0.026, U = 7.000, Z = −2.276), 3 days (4.71 ± 0.15, *P* = 0.007, U = 4.500, Z = −2.666), 7 days (4.21 ± 0.54, *P* = 0.017, U = 6.000, Z = −2.457), and 14 days (3.28 ± 0.57, *P* = 0.038, U = 8.500, Z = −2.620) after odor exposure. This heightened level of nociception was still observed 21 days later in animals subjected to all 3 risk factors when compared with naive levels (D29, 3.35 ± 0.47, *P* = 0.001, U = 1.000, Z = −3.050).

### 3.4. Repeated noninvasive vagus nerve stimulation and morphine administration transiently inhibited enhanced nociception

To test if nVNS and morphine could suppress the sustained nociception, animals subjected to the combined risk factors were treated daily with either nVNS or morphine for 7 consecutive days (days 11–17) starting 3 days after exposure to the odor (Fig. 5). Treatment with nVNS or morphine were similarly effective in significantly inhibiting the average number of nocifensive responses compared with chronic headache animals as seen 2 hours (nVNS: 0.5 ± 0.52, *P* = 0.007, U = 2.500, Z = −2.699; morphine: 0.25 ± 0.10, *P* = 0.001, U = 2.000, Z = −3.087) and 4 days (nVNS: 1.4 ± 0.45, *P* = 0.034, U = 6.500, Z = −2.115; morphine: 0.25 ± 0.06, *P* = 0.009, U = 6.000, Z = −2.642) post-treatment start. However, on day 19, which corresponds to one day after stopping nVNS or morphine treatment, the degree of nociception returned to pretreatment levels, which were significantly higher than naïve levels (nVNS: 4.5 ± 0.28, *P* = 0.002, U = 1.000, Z = −2.948, morphine: 4.4 ± 0.35, *P* = 0.002, U = 3.500, Z = −2.937) and remained significantly elevated at day 22 (nVNS: 3.7 ± 0.54, *P* = 0.035, U = 0.500, Z = −2.982; morphine: 4.2 ± 0.31, *P* = 0.001, U = 1.000, Z = −3.162) and day 29 (nVNS: 3.4 ± 0.49, *P* = 0.001, U = 0.500, Z = −2.982; morphine: 3.13 ± 0.42 *P* = 0.001, U = 1.000, Z = −3.162). These results demonstrate that nVNS and morphine can transiently inhibit trigeminal nociception to mechanical stimulation but are not able to reverse ongoing sensitization. One notable difference between the 2 treatments is that morphine resulted in a substantial reduction of responses to even the 180 g filament to below naïve levels, which was not observed with nVNS treatment (data not shown).

## 4. Discussion

A major finding of our study was that exposure of male animals to a combination of multiple migraine risks factors, rather than to each single risk factor, leads to an enhanced, prolonged hypersensitive trigeminal system characteristic of chronic headache.^22,38^ The 3 risk factors included inflammation of the trapezius,^13^ sleep deprivation,^61^ and exposure to the pungent odor from an oil extract of the California Bay Laurel tree, also known as the headache tree.^42^ Interestingly, animals subjected to a single risk factor did not exhibit a sustained increase in the average number of nocifensive responses to mechanical stimulation when compared with naive animals. However, inflammation in the trapezius, which was used as a model of neck muscle tension, or paradoxical sleep deprivation for one night, promoted latent trigeminal sensitization such that exposure to a nonpainful stimulus, a pungent odor, was sufficient to trigger an enhanced nocifensive response. The ability of the odorant to elicit trigeminal nociception in sensitized animals mimics the susceptibility of migraineurs to an attack by innocuous scents or odors.^52^

The extract used in our study contains umbellulone, which stimulates the release of calcitonin gene–related peptide from trigeminal neurons.^42^ Peripheral and central release of calcitonin gene–related peptide are implicated in the underlying pathology of migraine.^29^ Our findings are consistent with current theories that migraine pathology involves a reduction in the activity of the inhibitory pain pathway and development of a hyperexcitable or primed state of nociception.^22,30,43^ Based on results from other animal studies,^6,30,31,59^ neck muscle tension and sleep deprivation likely cause changes in descending pain modulation and an increased sensitivity of the trigeminal system to sensory stimuli such as lights, sounds, and odors that can trigger a migraine attack.^22,38^ The finding that trapezius inflammation mediates latent sensitization of the trigeminal system is in agreement with previous findings that exposure of sensitized animals to nitric oxide or to a pungent odor resulted in a transient increase in trigeminal nociception in models of episodic migraine.^15,24^ Tenderness and pain in the muscles of the head, neck, and shoulders are associated with migraine pathology and are commonly reported symptoms or comorbid conditions.^3,13,19,20,32^ This relationship is likely mediated by the convergence of nociceptive neurons that provide sensory innervation of neck and shoulder muscles and trigeminal nerves within the upper spinal cord.^7,41,49^

In this study, paradoxical or REM sleep deprivation was caused using a modified version of the platform method.^23,39^ This technique has been used to demonstrate that paradoxical sleep deprivation increases trigeminal pain levels by decreasing descending inhibitory pain modulation and/or by facilitating ascending pain signaling.^30,59^ For example, several nights of paradoxical sleep deprivation increased sensitivity of trigeminal nociceptive neurons to mechanical stimulation and increased c-Fos expression in the trigeminocervical complex, periaqueductal gray, and hypothalamus.^30^ Furthermore, sleep deprivation contributes to the development of facial allodynia, which is commonly reported by migraineurs during an attack and indicative of central sensitization.^9^ The combination of neck muscle inflammation and sleep deprivation resulted in a more prolonged level of trigeminal hypersensitivity that seemed to be resolving 7 days after sleep deprivation. However, exposure of these animals to the pungent odor resulted in a sustained elevation in nociception that remained significantly different than naive levels for at least 21 days. To the best of our knowledge, this is the first preclinical model of chronic headache that uses multiple migraine risk factors, neck muscle tension, and sleep deprivation, to mediate latent trigeminal nociceptor sensitization to a reported migraine trigger, a pungent odor. The sustained hypersensitivity of the trigeminal system seen in our model mimics a key feature of chronic headache, which is characterized by persistent central sensitization and a heightened pain response.^54^

In agreement with previous studies in which we demonstrated that nVNS inhibits trigeminal nociception in models of episodic migraine,^15,24^ results from this study support the notion that nVNS effectively suppresses trigeminal nociception in a model of chronic headache. Daily administration of nVNS significantly reduced the mechanical nocifensive response to naive levels. However, the inhibitory effect of nVNS was not sustained once treatments were ceased. This finding supports the approval of nVNS by the FDA as an acute treatment of migraine and episodic headache attacks in cluster headache patients^48^ and supports its potential benefit as an abortive therapeutic for chronic migraine.^63^

A similar pattern of inhibition of trigeminal nociception was observed with twice daily administration of morphine sulfate, a commonly used drug approved by the FDA for relief of moderate to severe pain. This is consistent with the ability of nVNS to inhibit nociception in models of migraine and other types of headache by stimulating descending inhibitory pain pathways known to be activated by morphine.^4,5,40^ More specifically, the inhibitory effect of nVNS involved activation of GABA_A_ receptors and 5-HT3 and 5-HT7 receptors in an episodic migraine model.^15^ Activation of GABA_A_ receptors on primary and second order neurons^1,37,55^ causes an influx of chloride ions that leads to a hyperpolarized state and blocks neurotransmitter and neuropeptide release. Noninvasive vagus nerve stimulation of the 5-HT3 and 5-HT7 receptors on inhibitory neurons results in activation of spinal interneurons and release of GABA and glycine to reduce pain signaling through the ascending pathway.

Activation of the serotonergic/GABAergic pathway is involved in the antinociceptive effects in other orofacial pain models.^56,57,62^ Noninvasive vagus nerve stimulation also inhibits elevated cerebrospinal fluid levels of the excitatory neurotransmitter glutamate in a nitroglycerin-induced model of trigeminal allodynia and thus may function to restore neurotransmitter homeostasis.^45^ Furthermore, our results provide evidence that nVNS and morphine only transiently suppressed trigeminal nociception because decreased nocifensive levels were no longer observed one day after stopping daily treatment with nVNS or morphine. This finding supports the notion of the persistent underlying pathology established in our chronic headache model. Although a single daily treatment of nVNS did not reverse ongoing hypersensitivity, one cannot rule out the possibility that multiple treatments per day, which is an accepted treatment paradigm in cluster headache patients,^63^ may reverse imbalances in pain modulation. Although nVNS functioned similarly to morphine to enhance serotonergic/GABAergic descending pain modulation, unlike morphine, nVNS is not associated with tolerance, hyperalgesia, or withdrawal.^48^

In summary, our findings provide evidence that the combination of neck muscle inflammation and REM sleep deprivation causes priming of the trigeminal system such that exposure to a pungent odor leads to a prolonged state of mechanical sensitivity/nociception characteristic of chronic headache in male animals. Hence, a potential limitation of our study is the inclusion of only male animals although chronic orofacial pain is more prevalent in women.^51^ Unfortunately, many chronic migraine patients do not get adequate relief with abortive antimigraine drugs including the triptans and opioids,^28,47^ so there is a need for improved therapies. Towards this end, our results demonstrate that nVNS suppressed ongoing trigeminal nociception to a similar level as morphine, which is commonly used in the emergency department to treat severe migraine if patients fail to respond to first-line and second-line therapies.^17,53^ Given the major public health concerns with opioids, the use of nVNS, which has proven beneficial in the treatment of other types of headache,^48^ may provide a safe and nonaddictive therapy for managing chronic trigeminally mediated pain conditions.

## Disclosures

The authors have no conflicts of interest to declare.
